# Relationship between Selected Trace Elements and Hematological Parameters among Japanese Community Dwellers

**DOI:** 10.3390/nu12061615

**Published:** 2020-05-30

**Authors:** Kyi Mar Wai, Kaori Sawada, Mika Kumagai, Kazuyoshi Itai, Itoyo Tokuda, Koichi Murashita, Shigeyuki Nakaji, Kazushige Ihara

**Affiliations:** 1 Department of Mibyo Science, Graduate School of Medicine, Hirosaki University, 5 Zaifu, Hirosaki 036-8562, Japan; nakaji@hirosaki-u.ac.jp (S.N.); ihara@hirosaki-u.ac.jp (K.I.); 2 Department of Social Medicine, Graduate School of Medicine, Hirosaki University, Hirosaki 036-8562, Japan; iwane@hirosaki-u.ac.jp; 3 Department of Active Life Promotion, Graduate School of Medicine, Hirosaki University, Hirosaki 036-8562, Japan; kumaga18@hirosaki-u.ac.jp; 4 Department of Nutritional Sciences, Morioka University, Iwate 020-0694, Japan; itai@morioka-u.ac.jp; 5 Department of Oral Health Care, Graduate School of Medicine, Hirosaki University, Hirosaki 036-8562, Japan; i-tokuda@hirosaki-u.ac.jp; 6 Center of Innovation, Research Initiatives Organization, Hirosaki University, Hirosaki 036-8562, Japan; murasita@hirosaki-u.ac.jp

**Keywords:** trace elements, metals, hematological parameters, anemia, Iwaki

## Abstract

This study aimed (1) to assess serum trace elements concentrations and hematological parameters, (2) to evaluate the sex differences in the associations between serum trace elements levels and hematological parameters, and (3) to identify the associations between serum trace elements concentrations and risk of anemia among Japanese community dwellers. This is a community-based cross-sectional study that utilized the data of the 2014 Iwaki Health Promotion Project. Participants were 1176 community dwellers (>18 years) residing in the Iwaki District, Aomori Prefecture, Japan. We assessed the data of serum trace elements concentrations of cadmium (Cd), cobalt (Co), copper (Cu), selenium (Se), zinc (Zn), and iron (Fe) as well as the hematological parameters of red blood cells (RBC) counts, hemoglobin, packed cells volume (PCV), mean corpuscular volume (MCV), and mean corpuscular hemoglobin (MCH). Serum concentrations of Zn (871.5 μg/L vs. 900.1 μg/L) and Fe (946.8 μg/L vs. 1096.1 μg/L) were significantly lower in females than in males, while serum concentrations of Co (0.4 μg/L vs. 0.3 μg/L) and Cu (1062.4 μg/L vs. 965.3 μg/L) were significantly higher in females. By multivariate linear regression, serum Se concentration was significantly, positively associated with PCV (β = 1.04; 95% confidence interval (CI): 0.17, 1.92; *p* = 0.016) among the study participants. Serum Zn also had positive associations with hemoglobin (β = 0.42; 95% CI: 0.07, 0.77; *p* = 0.020), PCV (β = 1.79; 95% CI: 0.78, 2.81; *p* < 0.001), and RBCs count (β = 15.56; 95% CI: 7.31, 31.69; *p* = 0.002). On the other hand, serum Co concentration was negatively associated with the hematological parameters, particularly in females. Moreover, serum Zn concentration had a decreased risk of anemia (lowest vs. highest quartiles: odds ratio (OR) = 0.42; 95% CI: 0.23, 0.76; *p* = 0.005) while higher Co concentrations had an increased risk of anemia (lowest vs. highest quartiles: OR = 1.95; 95% CI: 1.04, 3.67; *p* = 0.037). However, no significant association was found between serum Cu level and hematological parameters. There were substantial sex differences in serum trace elements, implying that trace elements metabolism differed between males and females. Zn can play a protective role in the development of anemia. Surprisingly, increased Co concentration increased the risk of anemia among our study population, which called for further studies to confirm and to consider for speciation analysis.

## 1. Introduction

Trace elements play an important role as essential components or cofactors of enzymes throughout hemopoiesis [1]. Most of the trace elements are critically involved during hemopoiesis via the metabolically important enzymatic pathway [1]. In addition, when the trace elements enter into the body, they may bind to red blood cells to be transported to target organs [2]. Thus, trace elements not only can alter the synthesis of red blood cells but also can influence the distribution and the storage of blood cells in the target organs, thereby altering the status of blood parameters in the body [2].

Among the several important trace elements, zinc (Zn), cobalt (Co), copper (Cu), and selenium (Se) are well known to be linked with hematological parameters. For example, Se is an essential trace element and major component of several selenoenzymes [3]. Previous studies demonstrated the link between the Se deficiency and the risk of anemia [4,5]. Zn also plays a critical role in nucleic acid metabolism, cellular replication, and repair [6]. Zn deficiency is one of the main contributing factors for anemia, since Zn assisted enzymes are essential to utilize Fe for hemoglobin synthesis [7]. In a previous study, serum Zn concentration was also found to be significantly lower in anemic individuals than in normal individuals [8]. Similarly, Co is consumed in cobalamin synthesis (vitamin B_12_), which is a crucial element of hemopoiesis [9]. Low Co concentration was reported to be associated with iron deficiency anemia [10]. In case of Cu, Cu acts as an essential component of the functioning enzymes such as ferroxidase, hephestin, and ceruloplasmin; it is related to the etiology of anemia due to defect in Fe immobilization [11,12]. In fact, trace elements, specifically Se, Cu, Zn, and Co, involve antagonistic or synergistic interactions, resulting in the alteration of the iron (Fe) delivery for hemopoiesis [13]. An established mechanism is the interaction of Fe and divalent metals at the intestinal transporters, disturbing the absorption and the excretion of Fe [13,14]. Thus, a deficiency of certain trace elements could be found in association with poor hematological parameters and anemia.

Imbalance of certain trace elements in the body not only influences the hematological parameters but also disturbs the absorption of other toxic elements. Cadmium (Cd) is considered as a xenobiotic metal whose chronic exposure may lead to severe health outcomes [15]. Once entered into the bloodstream, Cd appears to influence the storage and the absorption of other essential elements involved in hemopoiesis, particularly Fe and Zn [16,17]. Absorption of Cd is enhanced when Fe reserve in the body is low [16]. Several studies have also demonstrated that higher Cd concentration was associated with the anemia or low hematological parameters [2,16,18].

Anemia is a global public health concern, provoking severe health problems and lower quality of life [19,20,21]. According to the World Health Organization (WHO), anemia accounts for more than one-third of the world’s population [19]. The main contributory factor of anemia is the nutritional causes such as trace elements deficiency [20]. While the influence of sex on the risk of anemia is expected, there was only limited information that reported about the sex differences for trace elements concentrations in association with the various hematological parameters. Thus, the aims of this study were to assess serum trace elements concentrations and hematological parameters, to evaluate the sex differences in the associations between serum trace elements (Cd, Zn, Co, Cu, and Se) levels and hematological parameters, and to identify the associations between these serum trace elements concentrations and risk of anemia among Japanese community dwellers.

## 2. Methods

### 2.1. Study Design and Participants

This study was a cross-sectional design that utilized the data of the 2014 Iwaki Health Promotion Project. The Iwaki Health Promotion Project is an annual comprehensive health check-up among the residences in the Iwaki District of the Aomori Prefecture, Japan. The project was designated as a population-based study to assess the lifestyle and the health status of residents in Iwaki District. Participants were recruited through a public announcement, and the data collection was done in collaboration with Hirosaki University, Hirosaki City Office, and Aomori Prefectural General Screening Center. The study was approved by the research ethics committee of the Graduate School of Medicine, Hirosaki University (No. 2014-014). Prior to the participation, all subjects provided written informed consent.

In the 2014 Iwaki Health Promotion Project, a total of 1176 individuals aged 19 years and above were recruited. The present study excluded the individuals who did not complete questionnaire interviews or did not have trace elements and hematological data. This study also excluded the participants having a history of anemia and taking Fe supplements.

### 2.2. Hematological Parameters and Serum Trace Elements Measurement

Blood samples were collected from the participants from antecubital veins under the fasting condition. Serum separation was performed by centrifugation at 1000× g for 10 mins. Samples were stored at −80 °C until the laboratory experiments. In this study, we assessed the hematological parameters of hemoglobin, packed cell volume (PCV), mean corpuscular volume (MCV), mean corpuscular hemoglobin (MCH) and total red blood cells (RBC) counts. Compositions of blood were measured using an automated blood analyzer (Sysmex SE9000, Kobe, Japan).

Serum trace elements concentrations of Cd, Zn, Co, Cu and Se were measured using quadrupole, inductively coupled plasma mass spectrometry (ICP-MS) (SCIEX, ELAN6000, PerkinElmer Inc., Waltham, MA, USA). To prevent the contamination and disturbance, all the laboratory equipment in the trace elements measurement were washed with 15% nitric acid (Wako, Japan). The samples were measured against the multielement standard solution (TraceCERT, Sigma-Aldrich, LLC) by means of calibration curve. The measurements were externally validated against the certified reference material (Seronorm Trace Elements Serum L-1, Sero, Norway). All the values of trace elements measurements were within the acceptable ranges.

### 2.3. Covariates

Participants underwent an individual interview using questionnaires. The questionnaires covered the general characteristics of participants such as age, smoking, alcohol drinking, and medical history. Body composition was assessed using the Impedance Analyzer (Tanita Body Composition Analyzer MC180, Tokyo, Japan). Body mass index was then calculated according to the respective weight and height. Blood pressure was measured on the arm in sitting position by the trained staff using automatic blood pressure monitor (Omron Healthcare Co., Ltd., Kyoto, Japan), no later than 30 min of arrival to the health check-up center, approximately between 06:00 to 09:00. Fasting blood glucose level was assessed using the collected blood samples by the chemical reagent kit and biochemistry autoanalyzer (Iatoro LQ GLU, Unitica, Japan). Considering Fe status as an important factor of hemopoiesis, the serum Fe level was also included for the analysis as a covariate. Questionnaire survey, blood sampling, blood pressure, and body composition assessments were performed on the same day.

### 2.4. Statistical Analysis

Data were exported from Microsoft Excel, and statistical analysis was performed using Stata 13 (StataCorp LP, College Station, TX, USA). First, serum trace elements concentrations were expressed as median and interquartile range. To enhance the data distribution, trace elements concentrations were log-transformed or categorized into quartiles. Descriptive analysis was performed to explore the general characteristics of study subjects. Then, linear regression analysis was performed to examine the possible associations between trace element concentrations and hematological parameters with trace elements concentrations (log-transformed) as independent variables and hematological parameters (hemoglobin, PCV, MCV, MCH, and RBCs count) as dependent variables. According to WHO definition of anemia, two groups were categorized based on the hemoglobin concentration (1 = anemia, having hemoglobin concentration of <13 g% for males, and <12 g% for females; 0 = no anemia). Logistic regression was also performed to examine the risks of anemia in a relationship with trace element concentrations. The adjusted multivariate models included the confounders of age, sex, BMI, smoking, alcohol drinking, and serum Fe level. For all analyses, the significant level was set at a *p*-value of < 0.05.

## 3. Results

**Figure 1:**
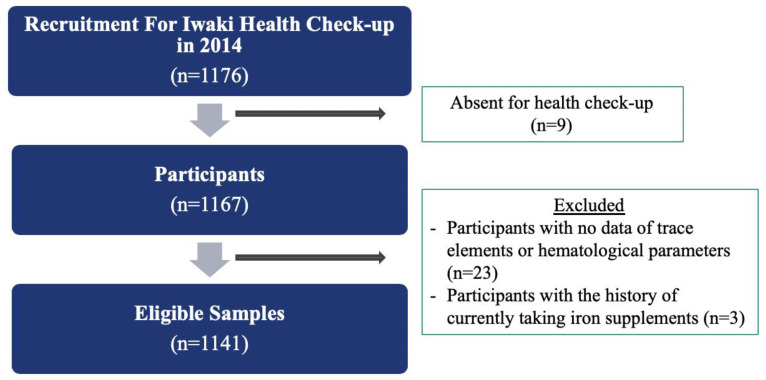
A flowchart for analytical samples.

Of the total recruitment of 1176 participants, only 1144 participants had complete data of the questionnaire survey, the trace elements concentration, or the hematological parameters. Of those, three participants were also excluded for currently taking Fe supplements (Figure 1). Thus, the analytical samples for the study were 1141 (433 males and 708 females).

General characteristics of participants are presented in Table 1. Participants were mostly middle-aged or old-aged people (median age = 57 years, interquartile range (IQR): 42–67 years) and non-smokers (64%). Among the study participants, the median BMI was 22.4 kg/m^2^ (IQR: 20.3–24.6 kg/m^2^) while the median blood sugar was 79.0 mg/dL (IQR: 74.0–87.0 mg/dL).

The mean concentrations of trace elements and hematological parameters are presented in Table 2. On stratification by sex, all the hematological parameters, i.e., hemoglobin, PCV, MCV, MCH, and RBCs, count were significantly lower in females. In addition, serum Zn (871.5 μg/L vs. 900.1 μg/L) and serum Fe (946.8 μg/L vs. 1096.1 μg/L) concentrations were also significantly lower in females, while Cu (1061.4 μg/L vs. 965.3 μg/L), serum Co (0.42 μg/L vs. 0.32 μg/L), and Cd (0.07 μg/L vs. 0.06 μg/L) concentrations were significantly higher in females. The prevalence of anemia in the study population was 12.1%. As expected, the proportion was higher among females (10.6%). The correlations between the trace elements are shown in Table 3. Significant negative associations were found between Fe-Cd pair (Spearman’s rho = −0.078, *p* = 0.012)), Fe-Cu pair (Spearman’s rho = −0.085, *p* = 0.004), and Fe-Co pair (Spearman’s rho = −0.169, *p* < 0.001), while a positive correlation was found between Fe-Se pair (Spearman’s rho = 0.079, *p* = 0.009) and Fe-Zn pair (Spearman’s rho = 0.086, *p* = 0.002).

Table 4 presents the results of multivariate linear regression models. After adjusting the confounders of age, sex, BMI, smoking status, alcohol drinking status, and serum Fe, a significant positive association was found between serum Se concentration level and PCV (β = 1.07; 95% CI: 0.19, 1.94). Particularly, in females, increased serum Zn showed significant positive associations with hemoglobin (β = 0.75; 95% CI: 0.27, 1.24), PCV (β = 2.54; 95% CI: 1.11, 3.89) and RBCs count (β = 23.65; 95% CI: 8.31, 38.98). On the other hand, serum Co concentration was negatively associated with all of the hematological parameters. No significant association was found between serum Cu level and hematological parameters in our study population. Among the confounders, BMI showed significant positive associations with hemoglobin, PCV, and RBCs count, while the hematological parameters declined with increasing age. In addition, to verify the general fact of the positive effect of Fe, the associations between serum Fe and hematological parameters were also assessed, and our study found no deviation.

Table 5 shows the associations between serum trace elements concentrations and risks of anemia by binary and multiple logistic regressions. Our study revealed that, compared to the lowest quartile group of serum Zn concentration, the highest Zn concentration group had about 60% decreased risks of anemia (Adjusted ORs = 0.42; 95% CI: 0.23, 0.76). On the other hand, surprisingly, the highest Co quartile was significantly associated with increased risks of anemia (Adjusted ORs = 1.95; 95% CI: 1.04, 3.67) among our study population.

**Figure 2:**
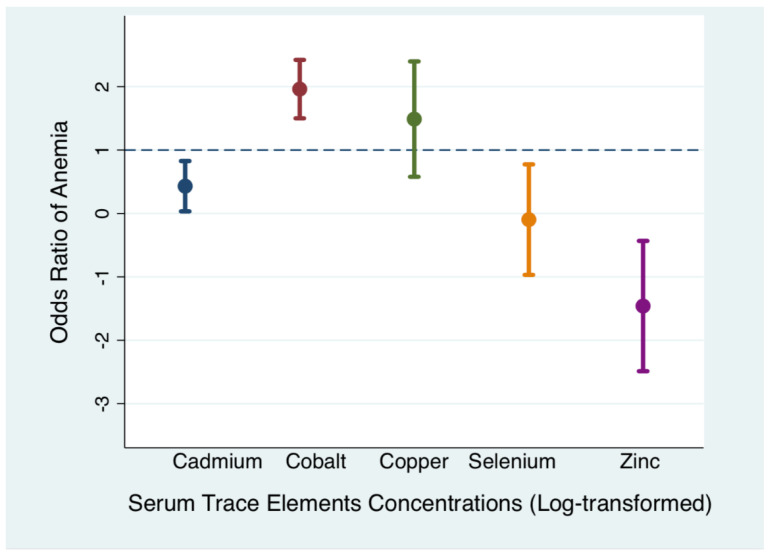
A regression plot to estimate the odds ratios (95% confidence intervals) of anemia by serum concentrations of cadmium, cobalt, copper, selenium, and zinc (*n* = 1141).

The odds ratios of anemia were also plotted using the continuous log-transformed values of serum trace elements concentrations as shown in Figure 2. Similarly, higher Zn concentration had a protective effect on anemia, while higher Cd and Co concentrations increased the risk of anemia.

## 4. Discussion

The current study revealed the important findings regarding the level of trace element concentrations in relation with different hematological parameters. There were female-specific positive associations between serum Zn concentration and hemoglobin, PCV, and RBCs counts. Meanwhile, regardless of sex, serum Co concentration had inverse associations with all hematological parameters (hemoglobin, PCV, MCV, MCH, and RBCs count). The risks of anemia increased by higher Co concentration, while it was protected by higher Zn concentration.

In this study, serum Zn concentration had a significant positive association with hemoglobin, PCV, and RBCs count. Moreover, the highest quartile of Zn was associated with a decreased risk of anemia. Literally, hematological parameters are well-reflected by the Fe status in the body. The previous findings on the effect of Zn on Fe status were controversial. For example, a trial study showed a higher concentration of Fe resulted in lowering the absorption of Zn in human adults, while another study reported that Zn absorption was not significantly different between the individuals who consumed iron-fortified food and those who consumed unfortified control food [6,7]. In fact, Fe acts as a major component of heme synthesis. In the case of Fe deficiency, Zn antagonizes the Fe absorption from the intestinal tract, resulting in lower serum Zn levels [22]. Moreover, several Zn-dependent enzymes facilitated hemopoiesis stimulation and hemoglobin synthesis [1]. It is also possible that micronutrient deficiencies of Zn and Fe co-exited as a nutritional imbalance. Therefore, our findings of the protective effects of Zn on anemia are plausible considering the above-mentioned facts.

Our study found significant inverse associations between serum Co concentration and hematological parameters. In other words, higher Co concentration increased the risk of anemia. In fact, Co is an essential component of vitamin B_12_ and other cobalamin enzymes. The results were unexpected while considering the role of Co in hemopoiesis through vitamin B_12_ synthesis [23]. However, a previous study supported our findings by reporting that increasing Co had a decrease in blood Fe of adolescents of both sex [13]. Another study among Norwegian women also demonstrated that Fe status showed a strong negative correlation with blood Co level [24]. Moreover, a significant inverse correlation was observed between the urinary Co level and hemoglobin concentrations in the third trimester pregnant women in Spain [25]. Increased intestinal absorption of Co is the consequence of the Fe depletion. Similar to other divalent metals, Co absorption is mediated by the divalent metal transporter 1 (DMT1), which is up-regulated by Fe status [26]. Thus, Fe depletion or higher Fe demand may trigger DMT1 expression, resulting in increased Co absorption.

In the current study, serum Se concentration was positively associated with PCV, although it showed no statistically significant association with the risk of anemia. Several studies reported the link between Se deficiency and anemia [4,27,28]. A possible biological evidence to support our finding is that Se may be involved in the regulation of hepatic heme oxygenase-1 activity [29]. Lower Se status can up-regulate the heme oxygenase-1 enzyme, which facilitates the heme catabolism, resulting in depletion of heme [29]. Another biological mechanism may be via the maintenance of glutathione peroxidase, which is a key selenoenzyme in RBCs [27]. The enzyme protects hemoglobin in RBCs from the oxidative damage and, thus, higher Se levels may result in increased PCV [27]. While low Se may contribute to the shortening of the half-life of RBCs and anemia, the current study was not very supportive, and it is necessary to confirm by well-designed intervention studies.

Serum Cd concentration showed no significant association with any of the hematological parameters in this study. In the previous studies, the increased Cd concentration was associated with Fe deficiency and lower levels of hematological parameters [2,16]. Being a divalent metal, Cd may interfere with Fe metabolism by competing at the DMT1 of the small intestine, resulting in a reverse relationship of Cd and Fe [30]. Furthermore, Cd may produce reactive oxygen species, which lead to the damage of RBC membrane and cellular injury [2]. On the contrary, the absence of significant association in this study could be explained by the lower concentration of Cd among the study population.

Similarly, our study revealed no significant association between serum Cu level and hematological parameters. This is rather surprising since there is a known biological relationship between Cu and Fe. Cu is a key component of ferroxidase enzyme, ceruloplasmin involved in the oxidation of ferrous to ferric ions. Approximately 90% of Cu in blood is bound to ceruloplasmin, and thus, depletion of Cu may impair Fe pharmacokinetics [11,12]. In accordance, a previous study also identified that lower level of blood Cu was found in anemic children compared to the normal healthy children [18], although this study showed no significant association. 

There were sex differences in the level of trace elements in the current study. In particular, serum Zn concentration in females was about 5% significantly lower, while serum Co concentration in females was about 30% higher than those observed in males. Although previous studies reported that females had lower concentrations of trace elements compared to males, the sex-specific relationship of their comparative lower concentration and hematological parameters was not clear [31,32]. The studies of trace elements and hematological parameters mostly emphasized the specific populations either at targeted age groups or specific cases such as pregnancy, anemia, and certain medical diseases [22,25,27,33]. As a strength, this study demonstrated that the associations between trace elements and hematological parameters were significantly influenced by sex among a general population. In the current study, females significantly experienced lower hematological parameters status from a higher concentration of Co or lower concentration of Zn. In fact, Zn and Fe deficiency can also coexist together, and Zn is also essential for hemoglobin synthesis [32]. In addition, it was demonstrated that females consumed less dietary Zn per day than that of males [32]. In the case of Co, Co intakes generally differed between males (20%) and females (45%) according to human biokinetic models considering the menstrual loss of Fe in females, which was associated with increased Co intake [34,35]. The fact was also supported by our finding that the serum ferritin level of the female participants in the study was approximately 2.5 times lower than that of males. Thus, sex-specific associations could be explained by the differences in the intake of Zn or Co as well as the differences in the level of Fe storage and Fe absorption in competing with the other trace elements.

In our study, significant negative correlations were found between the element pairs of Fe-Cd, Fe-Cu, and Fe-Co, while a positive correlation was found between Fe-Se and Fe-Zn pairs (Table 3). The results of significant correlation among the trace elements suggest the possibility that certain elements may mask the effect of other elements on the outcomes. However, the Spearman’s coefficients of element pairs were relatively small, indicating weak or very weak correlations except for the moderate correlation of Zn-Se pair. Therefore, the correlations between elements could not be objected, while their concealed effects on the hematological parameters was doubtful.

Some limitations should be considered in this study. The cross-sectional design precludes the interpretation of the causal relationships between trace elements and hematological parameters. Another potential limitation is the lack of consideration of dietary intakes that may have confounded our findings, since measuring circulating serum trace elements concentrations did not necessarily indicate the net intake [32]. The current study was also limited to identifying the correlations between individual trace elements and the blood pressure or sugar level. In fact, a lower Zn level was associated with a higher level of fasting blood sugar and glycated hemoglobin [36]. In addition, previous experimental and epidemiological studies identified that certain trace elements such as Cu, Cd, and Zn may influence blood pressure and cardiovascular dysfunctions [37,38]. Further analysis should be extended to examine the effects of trace elements on metabolic profiles including blood pressure and blood sugar level as main outcomes. Considering the biological actions of different species of trace elements, speciation analysis should also be performed.

## 5. Conclusions

Serum trace elements play a significant role in the hematological parameters, particularly in females. There are substantial sex differences in serum trace elements, implying that trace elements metabolism differs between males and females. Although sex differences in hematological parameters were expected, a particularly interesting finding of the current study was that the comparisons were made in relation to serum trace elements concentrations. Zn can play a protective role in the development of anemia. Surprisingly, higher Co concentration increased the risk of anemia among our study population, which called for further studies to confirm and to consider for speciation analysis.

## Figures and Tables

**Table 1:** General characteristics of participants (*n* = 1141).

Variables	Number	Frequency (%)	Median	[IQR]
**Age (years)**	1141		57	[42–67]
**Sex**	1141			
Male	433	37.9		
Female	708	62.1		
**Smoking Status**	1139			
Non-smokers	728	63.9		
Current Smokers	195	17.2		
Past Smokers	216	18.9		
**Alcohol Drinking**	1139			
Non-drinkers	604	53.1		
Current Drinkers	482	42.3		
Past Drinkers	53	4.6		
**Blood Pressure (mmHg)**	1141			
Systolic Blood Pressure			128	[115–143]
Diastolic Blood Pressure			77	[70–86]
**Body Mass Index (kg/m^2^)**	1141		22.4	[20.3–24.6]
**Fasting Blood Sugar (mg/dL)**	1141		79.0	[74.0–87.0]

IQR: Interquartile range. The bold is for easy reading.

**Table 2:** Sex differences in trace elements concentration and hematological parameters (*n* = 1141).

Variables	Male (*n* = 433)	Female (*n* = 708)	*p*-Value ^a^
**Serum Trace Elements Concentration (μg/L)**			
Cadmium	0.06	0.07	<0.001
Zinc	900.1	871.5	0.001
Cobalt	0.32	0.42	<0.001
Copper	965.31	1061.69	<0.001
Selenium	152.1	149.9	0.144
Iron	1096.1	946.8	<0.001
**Anemia**			<0.001
No	416 (36.5)	587 (51.5)	
Yes	17 (1.5)	121 (10.6)	
**Hematological Parameters**			
Hemoglobin (g/dL)	14.9	12.8	<0.001
PCV (%)	46.6	41.1	<0.001
MCV (fL)	97.3	95.2	<0.001
MCH (pg)	31.1	29.6	<0.001
RBCs Count (×10^4^ μg/L)	480.8	432.9	<0.001

PCV: packed cell volume; MCV: mean corpuscular volume; MCH: mean corpuscular hemoglobin; RBC: red blood cell. ^a^ *p-*values were derived from Mann–Whitney test for continuous variables and Chi-squared test for categorical variables. The bold is for easy reading.

**Table 3:** Correlation matrix of serum trace elements concentrations (Spearman’s rho, *n* = 1141).

Serum Trace Elements Concentration (μg/L)	Cadmium	Cobalt	Copper	Selenium	Zinc	Iron
Cadmium	1					
Cobalt	0.137 ***	1				
Copper	0.216 ***	0.120 ***	1			
Selenium	0.023	0.130 ***	0.288 ***	1		
Zinc	0.064 *	0.123 ***	0.157 ***	0.396 ***	1	
Iron	−0.078 **	−0.169 ***	−0.085 **	0.079 **	0.086 **	1

* *p* < 0.05; ** *p* < 0.01; *** *p* < 0.001.

**Table 4:** Associations between serum trace elements and hematological parameters stratified by sex (*n* = 1141).

Serum Trace Elements Concentrations (Log-Transformed)	Male (*n* = 433)		Female (*n* = 708)		All (*n* = 1141)	
	Beta ^†^	(95% CI)	Beta ^†^	(95% CI)	Beta ^‡^	(95% CI)
**Cadmium**						
Hemoglobin (g%)	−0.02	(−0.22, 0.19)	0.10	(−0.07, 0.27)	0.05	(−0.08, 0.18)
PCV (%)	−0.05	(−0.65, 0.55)	0.38	(−0.10, 0.86)	0.21	(−0.17, 0.58)
MCV (fL)	0.36	(−0.49, 1.22)	0.16	(−0.66, 0.98)	0.26	(−0.35, 0.88)
MCH (pg)	0.14	(−0.16, 0.43)	0.01	(−0.28, 0.32)	0.07	(−0.16, 0.29)
RBCs Count (×10^4^ μg/L)	−2.19	(−9.19, 4.80)	3.85	(−2.09, 9.81)	1.30	(−3.25, 5.86)
**Cobalt**						
Hemoglobin (g%)	−0.20	(−0.58, 0.19)	−0.53	(−0.75, −0.31) ***	−0.60	(−0.78, −0.41) ***
PCV (%)	−0.62	(−1.75, 0.52)	−1.17	(−1.79, −0.54) ***	−1.41	(−1.95, −0.88) ***
MCV (fL)	−0.71	(−2.51, 1.93)	−2.07	(−3.13, −1.02) ***	−2.29	(−3.15, −1.42) ***
MCH (pg)	−0.21	(−0.77, 0.35)	−1.02	(−1.39, −0.64) ***	−1.05	(−1.36, −0.74) ***
RBCs Count (×10^4^ μg/L)	−3.78	(−17.01, 9.53)	−1.87	(−10.42, 2.54)	−3.94	(−10.42, −0.30) *
**Copper**						
Hemoglobin (g%)	0.27	(−0.23, 0.77)	−0.10	(−0.52, 0.33)	−0.09	(−0.42, 0.24)
PCV (%)	1.21	(−0.23, 2.66)	0.12	(−1.05, 1.30)	0.18	(−0.76, 1.11)
MCV (fL)	1.60	(−0.52, 3.72)	−0.31	(−2.29, 1.67)	−0.13	(−1.62, 1.35)
MCH (pg)	0.19	(−0.55, 0.94)	−0.37	(−1.12, 0.38)	−0.37	(−0.93, 0.19)
RBCs Count (×10^4^ μg/L)	5.49	(−11.21, 22.19)	1.93	(−11.47, 15.33)	1.86	(−8.63, 12.36)
**Selenium**						
Hemoglobin (g%)	0.07	(−0.48, 0.55)	0.03	(−0.32, 0.39)	0.01	(−0.27, 0.33)
PCV (%)	1.33	(−0.05, 2.20)	0.80	(−0.22, 1.82)	1.04	(0.17, 1.92) *
MCV (fL)	0.66	(−1.32, 2.64)	1.06	(−0.66, 2.80)	0.50	(−0.83, 1.85)
MCH (pg)	−0.21	(−0.90, 0.48)	−0.17	(−0.80, 0.46)	−0.35	(−0.85, 0.13)
RBCs Count (×10^4^ μg/L)	5.23	(−11.02, 21.47)	3.42	(−9.23, 16.07)	5.28	(−4.67, 15.22)
**Zinc**						
Hemoglobin (g%)	0.69	(−0.50, 0.64)	0.58	(0.13, 1.01) **	0.42	(0.07, 0.77) **
PCV (%)	0.91	(−0.75, 2.57)	2.18	(0.92, 3.44) ***	1.79	(0.78, 2.81) ***
MCV (fL)	−0.47	(−2.85, 1.90)	−0.31	(−2.46, 1.84)	−0.27	(−1.92, 0.24)
MCH (pg)	−0.57	(−1.40, 0.25)	−0.29	(−1.08, 0.48)	−0.36	(−0.96, 0.24)
RBCs Count (×10^4^ μg/L)	11.09	(−8.37, 30.56)	23.86	(8.26, 39.46) **	19.50	(7.31, 31.69) **

PCV: packed cell volume; MCV: mean corpuscular volume; MCH: mean corpuscular hemoglobin; RBC: red blood cell; CI: confidence interval. ^†^ Adjusted for age, body mass index, smoking status, alcohol drinking status, and serum iron. ^‡^ Adjusted for sex, age, body mass index, smoking status, alcohol drinking status, and serum iron. * *p* < 0.05; ** *p* < 0.01; *** *p* < 0.001. The bold is for easy reading.

**Table 5:** Associations between serum trace elements concentration and risks of anemia (n = 1141).

Serum Trace Elements Concentration (μg/L)	Crude OR	(95% CI)	Adjusted OR ^†^	(95% CI)
**Cadmium**				
Quartile 1 (≤0.046)	ref		ref	
Quartile 2 (0.046–0.062)	1.30	(0.80, 2.34)	1.15	(0.61, 2.15)
Quartile 3 (0.062–0.081)	1.22	(0.71, 2.12)	0.81	(0.42, 1.55)
Quartile 4 (≥0.081)	1.86	(1.12, 3.12) *	1.05	(0.55, 1.97)
**Cobalt**				
Quartile 1 (≤0.29)	ref		ref	
Quartile 2 (0.29–0.34)	1.11	(0.58, 2.12)	0.87	(0.43, 1.73)
Quartile 3 (0.34–0.42)	1.64	(0.90, 2.98)	1.12	(0.59, 2.17)
Quartile 4 (≥0.42)	4.46	(2.60, 7.66) ***	1.95	(1.04, 3.67) *
**Copper**				
Quartile 1 (≤896.90)	ref		ref	
Quartile 2 (896.90–1004.00)	1.08	(0.60, 1.92)	0.83	(0.45, 1.55)
Quartile 3 (1004.00–1131.53)	2.18	(1.29, 3.67) **	1.72	(0.98, 3.03)
Quartile 4 (≥1131.53)	1.89	(1.10, 3.21) *	1.19	(0.66, 2.13)
**Selenium**				
Quartile 1 (≤129.86)	ref		ref	
Quartile 2 (129.86–148.50)	0.76	(0.46, 1.27)	0.78	(0.44, 1.38)
Quartile 3 (148.50–170.68)	0.97	(0.59, 1.58)	1.09	(0.63, 1.91)
Quartile 4 (≥170.68)	0.96	(0.69, 1.61)	1.17	(0.67, 2.04)
**Zinc**				
Quartile 1 (≤788.87)	ref		ref	
Quartile 2 (788.87–871.86)	0.53	(0.33, 0.86) *	0.52	(0.30, 0.89) *
Quartile 3 (871.86–965.82)	0.65	(0.41, 1.03)	0.86	(0.51, 1.46)
Quartile 4 (≥965.82)	0.34	(0.20, 0.58) ***	0.42	(0.23, 0.76) **

OR: odds ratio; CI: confidence interval. ^†^ Adjusted for age, sex, body mass index, smoking status, alcohol drinking status, and serum iron. * *p* < 0.05; ** *p* < 0.01; *** *p* < 0.001. The bold is for easy reading.
